# Five-Year Follow-Up of Work Disability After Traumatic Brain Injury: A Nationwide Swedish Matched Cohort Study of 98,000 Individuals

**DOI:** 10.1212/WNL.0000000000214674

**Published:** 2026-02-11

**Authors:** Andrea Klang, Yasmina Molero, Jakob Bergström, Ellenor Mittendorfer-Rutz, Christian Oldenburg, Elham Rostami

**Affiliations:** 1 Rehabilitation Medicine, Department of Medical Sciences, Uppsala University, Sweden;; 2 Department of Clinical Neuroscience, Karolinska Institutet, Stockholm, Sweden;; 3 Department of Medical Epidemiology and Biostatistics, Karolinska Institutet, Stockholm, Sweden;; 4 Division of Insurance Medicine, Department of Clinical Neuroscience, Karolinska Institutet, Stockholm, Sweden;; 5 Neurosurgery, Department of Medical Sciences, Uppsala University, Sweden; and; 6 Department of Neuroscience, Karolinska Institutet, Stockholm, Sweden.

## Abstract

**Background and Objectives:**

Traumatic brain injury (TBI) is a leading cause of long-term disability in working-age populations. Return to work is a key marker of recovery, yet most studies assess it as binary at fixed time points. We aimed to estimate transition probabilities to and from work disability during 5 years after TBI and how injury severity and preinjury sociodemographic and medical factors influence these probabilities.

**Methods:**

We conducted a nationwide matched cohort study in Sweden using linked registers. Individuals aged 21–60 years with a TBI diagnosis between 2005 and 2016 were compared with up to 10 matched non-TBI individuals. TBI severity was proxied by care characteristics: TBI A (emergency visit or ≤2 days), TBI B (≥3 days), and TBI C (neurosurgery). Transition probabilities to and from work disability (>14 days sickness absence) were estimated with multistate models. Sociodemographic and medical factors were assessed with Cox regression.

**Results:**

The cohort included 98,256 individuals with TBI and 981,191 matched non-TBI individuals (median age 39 years; 43% women). Transition probabilities to work disability were higher in all TBI groups: at 30 days, 5.5% (95% CI 5.4–5.7) for TBI A, 29% (28.0–30.7) for TBI B, and 43% (38.2–47.3) for TBI C, vs 0.5% (0.5–0.6) in non-TBI; at 5 years, 7.1% (7.0–7.3), 10.9% (10.2–11.7), and 12.9% (10.7–15.7), vs 4.0% (4.0–4.1). In TBI A and B, higher probability was predicted by older age (TBI A hazard ratio 1.23, 95% CI 1.20–1.26; TBI B 1.34, 1.21–1.48), female sex (TBI A 1.59, 1.56–1.62; TBI B 1.35, 1.26–1.44), and psychiatric disorders (TBI A 1.34, 1.30–1.39; TBI B 1.28, 1.11–1.48), while higher education (TBI A 0.83, 0.81–0.86) and city residence (TBI A 0.92, 0.90–0.95; TBI B 0.88, 0.80–0.95) were protective. In TBI C, only older age remained significant (1.59, 1.17–2.14).

**Discussion:**

TBI was associated with persistently elevated transition probabilities to work disability across all severity groups, with early peaks in TBI B and C and a delayed increase in TBI A, influenced by sociodemographic and medical factors. However, the lack of standardized severity grading limits comparison with other studies. Still, these results suggest TBI increases long-term risk of work disability, supporting sustained individualized rehabilitation.

## Introduction

Traumatic brain injury (TBI) is a major cause of mortality and disability in working-age populations^1^ Each year, 50–60 million people sustain a TBI, and the incidence of hospital-treated TBIs in Europe is about 287 per 100,000 person-years.^1^ Most TBIs are mild, yet recovery is often incomplete and long-term consequences remain poorly understood.^2^ Nearly all patients with severe TBI experience lasting symptoms that affect function and daily life.^3^

Return to work is an important outcome for working-age patients with TBI, benefiting well-being, quality of life, and financial stability.^4^ It also reduces societal costs and allows employers to regain productive workers.^5^ Previous studies have shown varying rates of inability to return to work after a TBI, where 5%–29% of mild patients with TBI had not returned to work within 6–12 months.^6,7^ For patients with moderate-to-severe TBI, work disability is higher, and after a few years, only 44%–55% were employed.^8,9^ Few consistent predictors of return to work have been identified in moderate-to-severe TBI, although preinjury employment, occupation type, injury severity, and hospital stay length are relevant.^9,10^ In mild TBI, education and indirect severity measures (e.g., nausea, extracranial injuries) have been linked to work outcomes.^6,7^ Still, evidence remains inconsistent regarding sociodemographic factors such as sex, age, psychiatric and substance use disorders, and prior work disability.^6,7,9,10^ Long-term studies also face methodologic limitations, including biased recruitment,^11^ lack of controls,^3,6^ small samples,^6,9^ and loss to follow-up,^12^ which reduce robustness and generalizability.

TBI is increasingly recognized as a chronic condition with enduring symptoms and fluctuating recovery.^13^ Individuals may recover spontaneously or through interventions,^14,15^ but may also deteriorate due to trauma-related consequences.^16^ Outcomes are often assessed only 1–2 years postinjury and return to work is typically treated as a binary end point.^6,10^ This approach fails to capture the dynamic process of work disability over time, with transitions in and out of disability influenced by sociodemographic, medical, and injury-related factors.^17^

To address these methodologic challenges, we propose using a large longitudinal cohort that includes individuals treated for all TBI severities, with follow-up over several years in nationwide complete registers. This approach ensures minimal attrition and balanced geographical representation, which has not been comprehensively achieved across all TBI severities in previous studies.^18^ Furthermore, by incorporating a matched cohort without TBI, work disability transitions can be compared, and preinjury factors associated with work disability can be systematically analyzed. The use of a multistate model allows for tracking transitions between work disability and nonwork disability as recurrent events, while incorporating patient-specific factors as predictors of these transitions. In this way, the multistate model provides a more nuanced understanding of the multiple pathways after a TBI compared with traditional methods.

This study uses Swedish nationwide registers to compare a population-based TBI cohort with a matched cohort of individuals without TBI. Our primary aim was to examine the probability of transitioning between work disability and nonwork disability for up to 5 years postinjury, by comparing individuals with TBI with those without TBI and using a multistate model. Furthermore, we aimed to investigate patient-specific sociodemographic and health-related factors associated with the risk of work disability after a TBI.

## Methods

### Study Design

This was a population-based longitudinal study in Sweden, linking several nationwide high-quality registers, including the Micro-Data for Analysis of the Social Insurance System (MiDAS), National Patient Register (NPR), Total Population Register, Longitudinal Integrated Database for Health Insurance and Labor Market Studies, and the Cause of Death register.

### Study Population and Procedures

We included all individuals with a TBI diagnosis (main or secondary diagnosis) recorded in specialized health care, that is, hospital and specialized outpatient care and emergency departments, from the NPR during 2005–2016. Inclusion was limited to individuals aged 21–60 years at the time of injury, allowing 3 years of prior data and 5 years of follow-up, within the legal adult age of 18 years and retirement age of 65 years during the study period.^19^ We excluded individuals with a TBI diagnosis during the 3 years before 2005, to ensure incident TBI. To approximate TBI severity, patients were classified into 3 hierarchical and mutually exclusive proxy groups based on care characteristics:

TBI A: specialized health care visit or hospital stay of ≤2 days (maximum 1 night of observation), without neurosurgical procedure.
TBI B: hospital stay of ≥3 days, without neurosurgical procedure.
TBI C: neurosurgical procedure indicative of severe trauma.

These categories serve as proxies for severity in the absence of standardized clinical data such as the Glasgow Coma Scale. Every patient with TBI was matched with up to 10 non-TBI individuals, conditioning on survival at the end of the year of TBI diagnosis. For detailed information, see eMethods.

Primary outcome event was transition probability to or from work disability, defined as a period of >14 net days (i.e., whole days) of sickness absence and/or disability pension during the follow-up (5 years in total), as in previous research,^20^ as registered in MiDAS.^21^ In Sweden, all residents of working age with income are covered by the national social insurance system and thus eligible for sickness absence benefits. Individuals could have reoccurring periods of work disability with intervening periods of nonwork disability. In this context, nonwork disability refers to not receiving work disability benefits; it may include individuals who returned to work or studies as well as those outside the labor market without ongoing benefit entitlement. Thus, the outcome reflects insurance-based work disability status rather than direct employment status. Sociodemographic and medical data were collected from national registers and used for baseline characteristics and covariates, measured up to 3 years preinjury (see eMethods).

### Statistical Analyses

A reversible multistate model using Cox regression was used to estimate instantaneous hazards and probabilities of transitioning between the transient states nonwork disability and work disability, eFigure 1. Cohort entry date was defined as the date of TBI diagnosis for cases. The same index date was used for the references matched to the case. Follow-up started on day 0 and ended on day 1,825 (i.e., 5 years after). Individuals could start follow-up in either a work disabled or nonwork disabled state. The process stopped if an individual was alive at end of follow-up or had transitioned to the absorbing state of death or migration. The competing events death and migration were analyzed as cause-specific, that is, they were treated as separate outcome events.

Predictors for transitions between nonwork disability and work disability were assessed using Cox regression adjusted for baseline sociodemographic factors (age, sex, education, occupational status, living area, family status, prior work disability, and income from work or studies) and baseline comorbidities (cancer, diabetes, cardiovascular disease, psychiatric and substance use disorders, and co-occurring injuries), see Table 1 footnote and eMethods for details. Hazard ratios (HRs) and 95% CIs are reported for all analyses. To avoid immortal time bias, we used a matched cohort design ensuring comparable follow-up periods. The TBI groups were analyzed in separate models because of violation of the proportional hazard's assumption. All individuals were stratified by being in a period with work disability state, or not, at baseline. First, we compared the proportion of individuals who transitioned at least once from nonwork disability to work disability in each TBI group and the matched cohort, using logistic regression to estimate the risk ratio. We repeated the same analyses for individuals who started in work disability at baseline and transitioned at least once to nonwork disability.

**Table 1:** Sociodemographic and Preinjury Medical Factors of the Cohort at Baseline (i.e., at the Time of the TBI Diagnosis)

	Total (N = 1,079,447)	Non-TBI (N = 981,191)	TBI A (N = 91,505)	TBI B (N = 6,091)	TBI C (N = 660)
Sex^a^					
Women	454,683 (42.12)	413,291 (42.12)	39,228 (42.87)	1,979 (32.49)	185 (28.03)
Men	624,764 (57.88)	567,900 (57.88)	52,277 (57.13)	4,112 (67.51)	475 (71.97)
Age group, y^a^					
21–30	349,246 (32.35)	317,447 (32.35)	30,647 (33.49)	993 (16.30)	159 (24.09)
31–40	228,943 (21.21)	208,110 (21.21)	19,807 (21.65)	910 (14.94)	116 (17.58)
41–50	250,090 (23.17)	227,325 (23.17)	21,008 (22.96)	1,601 (26.28)	156 (23.64)
51–60	251,168 (23.27)	228,309 (23.27)	20,043 (21.90)	2,587 (42.47)	229 (34.70)
Age, y, median (Q1–Q3)	39.0 (27.0–50.0)	39.0 (27.0–50.0)	38.0 (27.0–49.0)	48.0 (37.0–55.0)	45.0 (32.0–54.0)
Country of birth^a^					
Sweden	885,335 (82.02)	804,825 (82.03)	74,811 (81.76)	5,147 (84.50)	552 (83.64)
Europe without Sweden	61,575 (5.70)	55,920 (5.70)	5,228 (5.71)	384 (6.30)	43 (6.52)
Other	132,537 (12.28)	120,446 (12.28)	11,466 (12.53)	560 (9.19)	65 (9.85)
Level of education^a^					
0–9 y	212,520 (19.69)	193,168 (19.69)	17,787 (19.44)	1,422 (23.35)	143 (21.67)
10–12 y	557,704 (51.67)	506,969 (51.67)	47,244 (51.63)	3,126 (51.32)	365 (55.30)
13- y	309,223 (28.65)	281,054 (28.64)	26,474 (28.93)	1,543 (25.33)	152 (23.03)
Living area^a^					
Rural	199,294 (18.46)	181,172 (18.46)	16,674 (18.22)	1,346 (22.10)	102 (15.45)
Towns and suburbs	447,183 (41.43)	406,488 (41.43)	37,913 (41.43)	2,549 (41.85)	233 (35.30)
Cities	432,970 (40.11)	393,531 (40.11)	36,918 (40.35)	2,196 (36.05)	325 (49.24)
Family status^a,b^					
Married or cohabitant without children	177,639 (16.46)	165,020 (16.82)	11,328 (12.38)	1,200 (19.70)	91 (13.79)
Married or cohabitant with children	309,736 (28.69)	286,662 (29.22)	21,894 (23.93)	1,058 (17.37)	122 (18.48)
Single without children	542,554 (50.26)	486,085 (49.54)	52,526 (57.40)	3,520 (57.79)	423 (64.09)
Single with children	49,518 (4.59)	43,424 (4.43)	5,757 (6.29)	313 (5.14)	24 (3.64)
Prior work disability^c^					
No	958,067 (88.76)	878,778 (89.56)	74,388 (81.29)	4,384 (71.98)	517 (78.33)
Yes	121,380 (11.24)	102,413 (10.44)	17,117 (18.71)	1,707 (28.02)	143 (21.67)
Current income from work or studies^a^					
No	228,880 (21.20)	205,918 (20.99)	20,777 (22.71)	1,980 (32.51)	205 (31.06)
Yes	850,567 (78.80)	775,273 (79.01)	70,728 (77.29)	4,111 (67.49)	455 (68.94)
Occupational status^a^					
White collar	322,444 (29.87)	295,416 (30.11)	25,174 (27.51)	1,674 (27.48)	180 (27.27)
Blue collar	400,094 (37.06)	358,378 (36.52)	38,985 (42.60)	2,454 (40.29)	277 (41.97)
Unknown	356,909 (33.06)	327,397 (33.37)	27,346 (29.88)	1,963 (32.23)	203 (30.76)
Cancer^c^					
No	1,049,988 (97.27)	954,262 (97.26)	89,192 (97.47)	5,893 (96.75)	641 (97.12)
Yes	29,459 (2.73)	26,929 (2.74)	2,313 (2.53)	198 (3.25)	19 (2.88)
Diabetes^c^					
No	1,067,156 (98.86)	970,429 (98.90)	90,203 (98.58)	5,881 (96.55)	643 (97.42)
Yes	12,291 (1.14)	10,762 (1.10)	1,302 (1.42)	210 (3.45)	17 (2.58)
Cardiovascular disorder^c^					
No	1,047,917 (97.08)	953,901 (97.22)	87,913 (96.07)	5,506 (90.40)	597 (90.45)
Yes	31,530 (2.92)	27,290 (2.78)	3,592 (3.93)	585 (9.60)	63 (9.55)
Psychiatric disorder^c^					
No	1,046,428 (96.94)	953,450 (97.17)	86,643 (94.69)	5,712 (93.78)	623 (94.39)
Yes	33,019 (3.06)	27,741 (2.83)	4,862 (5.31)	379 (6.22)	37 (5.61)
Substance use disorder^c^					
No	1,066,056 (98.76)	972,739 (99.14)	87,144 (95.23)	5,581 (91.63)	592 (89.70)
Yes	13,391 (1.24)	8,452 (0.86)	4,361 (4.77)	510 (8.37)	68 (10.30)
Co-occurring body injuries^d^					
No	1,051,355 (97.40)	980,834 (99.96)	66,777 (72.98)	3,331 (54.69)	413 (62.58)
Yes	28,092 (2.60)	357 (0.04)	24,728 (27.02)	2,760 (45.31)	247 (37.42)

Abbreviations: LISA = Longitudinal Integrated Database for Health Insurance and Labor Market Studies; TBI = traumatic brain injury.

Presented as N (%).

a Sociodemographic data were recorded the last of November before the injury (or index date for non-TBI). Occupational status was obtained from the LISA register and classified as white collar (professional/administrative) or blue collar (manual labor) using the Swedish Standard Classification of Occupations (SSYK, aligned with ISCO). Living area was measured according to DEGURBA. Details in eMethods.

b Family status measured as children <18 years old living at home.

c Presence of preinjury work disability (sick leave longer than >14 days or disability pension) and comorbidities were measured during 3 years before the TBI. Comorbidities were measured as the presence of specified ICD-10 from specialized health care. Details in eMethods.

d Specified ICD-10 codes, measured at the date of the TBI diagnosis and during the entire hospital stay if inpatient care.

Data for the main diagnosis of work disability on the medical certificate were collected from MiDAS (eTable 1). Average mean time in each diagnosis category was calculated by restricted mean time survival analysis, see eMethods.

Between 2008 and 2015, Swedish social insurance regulations introduced a maximum entitlement of 914 days of sickness benefit, after which individuals were required to reapply or transition to other forms of support.^22^ This reform affected the long-term measurement of work disability, and we therefore performed sensitivity analyses excluding this period (see eMethods). The same reverse multistate model and Cox regression analysis were performed for this subcohort.

R version 4.4.1 with the package mstate (version 0.3.3) was used to estimate the multistate model. Data management was performed using SAS version 9.4 and Stata 17.0. The funder of the study had no role in study design, data collection, data analysis, data interpretation, or writing of the report. The Strengthening the Reporting of Observational studies in Epidemiology reporting guidelines were followed.

### Standard Protocol Approvals, Registrations, and Patient Consents

This study followed the Declaration of Helsinki and was approved by the Swedish Ethical Review Authority (reference numbers 2007/762-31 and 2021-06441-02). Given the retrospective register-based design, the requirement for informed consent was waived by the Ethical Review Authority.

### Data Availability

The data used in this study cannot be made publicly available because of privacy regulations. According to the General Data Protection Regulation, the Swedish law SFS 2018:218, the Swedish Data Protection Act, the Swedish Ethical Review Act, and the Public Access to Information and Secrecy Act, these types of sensitive data can only be made available for specific purposes, including research, that meets the criteria for access to this sort of sensitive and confidential data as determined by a legal review. Readers may contact Professor Ellenor Mittendorfer-Rutz (ellenor.mittendorfer-rutz@ki.se) regarding the data.

## Results

The final cohort included 98,256 individuals with TBI and 981,191 without TBI. TBI A (≤2 days, no neurosurgery) comprised 93% (n = 91,505), TBI B (≥3 days, no neurosurgery) 6% (n = 6,091), and TBI C (neurosurgery) <1% (n = 660). The median age ranged from 38 years in TBI A (interquartile range [IQR] 27–49) to 48 years in TBI B (IQR 37–55), and men constituted the majority in all groups (57%–72%) (Table 1). Compared with non-TBI, individuals with TBI more often lived alone, had preinjury work disability and comorbidities (cardiovascular disease, diabetes, psychiatric disorders), and less often had income from work or studies (Table 1).

**Figure 1:**
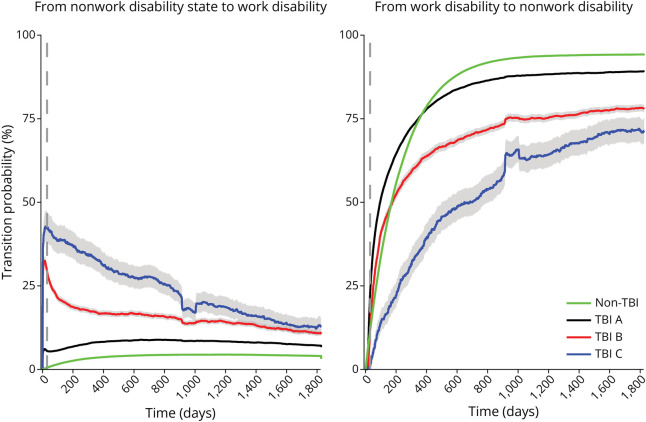
Transition Probabilities to and From Work Disability TBI = traumatic brain injury.

Transition probabilities from nonwork disability (not receiving benefits) to work disability (receiving benefits) were consistently higher in all TBI groups than in non-TBI. In TBI B and C, probabilities peaked early (30 days: 29% and 43%) and then declined to 12%–13% at 5 years (Figure 1 and eTable 2). In TBI A, probability increased gradually, stabilizing at 9% by 2 years compared with 4% in non-TBI. Transitions from work disability to nonwork disability followed the reverse pattern, with the lowest recovery probability in TBI C (Figure 1).

During follow-up, the proportion experiencing at least 1 episode of work disability increased with severity: 26% in non-TBI, 45% in TBI A, 67% in TBI B, and 72% in TBI C. Excluding those with prior work disability yielded lower proportions but the same pattern (Table 2).

**Table 2:** At Least 1 Period of Work Disability During the Follow-Up Period of 5 Years

	Non-TBI (reference)	TBI A	TBI B	TBI C
At least 1 period of work disability	26 (254,625)	45 (40,875)	67 (4,080)	72 (472)
Risk ratio	1	1.72 (1.71–1.74)	2.58 (2.53–2.63)	2.76 (2.62–2.89)
Risk ratio. Adjusted for baseline covariates^a^	1	1.66 (1.65–1.68)	2.63 (2.57–2.69)	2.98 (2.82–3.15)
At least 1 period of work disability given no work disability at baseline	23 (212,082)	35 (27,587)	44 (1,561)	51 (196)
Risk ratio given no work disability at baseline	1	1.56 (1.55–1.58)	1.93 (1.86–2.01)	2.26 (2.04–2.48)
Risk ratio given no work disability at baseline. Adjusted for baseline covariates^a^	1	1.55 (1.53–1.57)	2.11 (2.03–2.19)	2.54 (2.30–2.78)

Abbreviation: TBI = traumatic brain injury.

Proportions% (n) and risk ratios with 95% CIs.

a Predictors are the same baseline sociodemographic factors and comorbidities described in Methods and detailed in Table 1 footnote (see eMethods for ICD-10 codes).

In TBI A and B, positive predictors of transitioning to work disability included older age (TBI A HR 1.23, 95% CI 1.20–1.26; TBI B HR 1.34, 1.21–1.48), female sex (TBI A 1.59, 1.56–1.62; TBI B 1.35, 1.26–1.44), blue-collar (manual/industrial) occupation (TBI A 1.31, 1.29–1.34; TBI B 1.22, 1.13–1.33), psychiatric disorders (TBI A 1.34, 1.30–1.39; TBI B 1.28, 1.11–1.48), substance use disorders (TBI A 1.32, 1.26–1.38; TBI B 1.20, 1.04–1.38), income from work or studies (TBI A 2.30, 2.23–2.36; TBI B 3.54, 3.17–3.96), and prior work disability (TBI A 1.39, 1.36–1.42). Negative predictors included higher education (TBI A 0.83, 0.81–0.86) and city residence (TBI A 0.92, 0.90–0.95; TBI B 0.88, 0.80–0.95). In TBI C, older age was the only consistent risk factor (1.59, 1.17–2.14). Individuals with income from work or studies had a higher risk of transitioning to work disability (all groups HR 2.26–5.84) while only in TBI C, those with prior work disability had a lower risk (0.63, 0.44–0.91). Individuals in the non-TBI cohort presented a similar overall pattern of sociodemographic and medical predictors as in TBI A (Table 3).

**Table 3:** Predictors for Transitioning From Nonwork Disability to Work Disability During the 5-Year Follow-Up

Covariate	Levels	Non-TBI	TBI A	TBI B	TBI C
		HR (95% CI)	HR (95% CI)	HR (95% CI)	HR (95% CI)
Age, y	21–30	1	1	1	1
	31–40	1.16 (1.15–1.17)	1.12 (1.10–1.15)	1.12 (1.00–1.26)	0.85 (0.62–1.17)
	41–50	1.22 (1.20–1.23)	1.13 (1.10–1.15)	1.08 (0.98–1.20)	1.40 (1.04–1.90)
	51–60	1.43 (1.42–1.45)	1.23 (1.20–1.26)	1.34 (1.21–1.48)	1.59 (1.17–2.14)
Sex	Men	1	1	1	1
	Women	1.78 (1.76–1.79)	1.59 (1.56–1.62)	1.35 (1.26–1.44)	1.14 (0.92–1.43)
Level of education	0–9 y	1	1	1	1
	10–12 y	0.87 (0.86–0.88)	0.98 (0.96–1.00)	1.03 (0.95–1.13)	0.90 (0.70–1.17)
	13- y	0.71 (0.70–0.72)	0.83 (0.81–0.86)	1.04 (0.93–1.15)	0.91 (0.65–1.26)
Living area	Rural	1	1	1	1
	Towns and suburbs	0.98 (0.97–0.99)	1.00 (0.98–1.02)	0.97 (0.89–1.05)	0.94 (0.70–1.26)
	Cities	0.94 (0.93–0.95)	0.92 (0.90–0.95)	0.88 (0.80–0.95)	1.01 (0.76–1.35)
Family status	Married/cohabitant without children	1	1	1	1
	Married or cohabitant with children	1.01 (1.00–1.02)	1.00 (0.97–1.03)	1.03 (0.93–1.14)	1.40 (0.98–2.01)
	Single without children	0.98 (0.97–0.99)	0.95 (0.92–0.97)	1.02 (0.94–1.12)	1.25 (0.91–1.73)
	Single with children	1.28 (1.26–1.30)	1.25 (1.21–1.30)	1.23 (1.07–1.42)	1.54 (0.88–2.69)
Occupational status	White collar	1	1	1	1
	Blue collar	1.46 (1.45–1.47)	1.31 (1.29–1.34)	1.22 (1.13–1.33)	1.22 (0.94–1.58)
	Unknown	0.94 (0.93–0.95)	0.88 (0.85–0.90)	0.80 (0.72–0.90)	1.09 (0.78–1.50)
Current income from work or studies	No	1	1	1	1
	Yes	2.26 (2.23–2.28)	2.30 (2.23–2.36)	3.54 (3.17–3.96)	5.84 (4.16–8.21)
Country of birth	Sweden	1	1	1	1
	Europe other than Sweden	1.06 (1.04–1.07)	1.07 (1.03–1.11)	1.00 (0.87–1.15)	0.61 (0.39–0.97)
	Other	1.04 (1.02–1.05)	1.09 (1.06–1.12)	1.08 (0.96–1.21)	0.47 (0.30–0.73)
Prior work disability	No	1	1	1	1
	Yes	1.66 (1.65–1.68)	1.39 (1.36–1.42)	0.92 (0.84–1.01)	0.63 (0.44–0.91)
Cancer	No	1	1	1	1
	Yes	1.14 (1.12–1.16)	1.05 (1.00–1.10)	1.12 (0.94–1.33)	1.05 (0.53–2.06)
Diabetes	No	1	1	1	1
	Yes	1.43 (1.40–1.47)	1.30 (1.22–1.38)	1.08 (0.88–1.33)	0.61 (0.30–1.27)
Cardiovascular disorder	No	1	1	1	1
	Yes	1.32 (1.30–1.35)	1.31 (1.26–1.36)	0.99 (0.87–1.12)	1.26 (0.88–1.82)
Psychiatric disorder	No	1	1	1	1
	Yes	1.46 (1.43–1.49)	1.34 (1.30–1.39)	1.28 (1.11–1.48)	0.87 (0.50–1.51)
Substance use disorder	No	1	1	1	1
	Yes	1.67 (1.62–1.73)	1.32 (1.26–1.38)	1.20 (1.04–1.38)	1.19 (0.82–1.75)
Co-occurring body injuries	No	1	1	1	1
	Yes	1.90 (1.67–2.16)	1.04 (1.02–1.06)	1.21 (1.14–1.29)	1.03 (0.84–1.27)

Abbreviations: HR = hazard ratio; TBI = traumatic brain injury.

Presented with HR (95% CI).

Predictors are the same baseline sociodemographic factors and comorbidities described in Methods and detailed in Table 1 footnote (see eMethods for ICD-10 codes).

In a second set of analyses, we examined factors influencing the transition from work disability at index to nonwork disability (i.e., not receiving benefits). Most factors had the opposite association compared with the previous analyses, with a few exceptions: female sex (TBI A 1.02, 1.00–1.03) and blue-collar work (TBI A 1.13, 1.10–1.15; TBI B 1.10, 1.02–1.18) (eTable 3).

On average, days with work disability increased with severity, from 526 days in TBI A to 1,201 in TBI C during 5 years. The most common cause in TBI A and B and non-TBI was miscellaneous diagnoses (i.e., a broad category including specific conditions such as cancer as well as symptom-based conditions like pain and fatigue). In TBI C, TBI-related diagnoses were the leading cause, accounting for 40% of work disability (Table 4, eTable 1).

**Table 4:** Average Time in Days for Different Causes of Work Disability^a^ During the 5-Year Follow-Up, Based on Restricted Mean Survival Time Analysis

ICD-10 group	Non-TBI	TBI A	TBI B	TBI C
	Mean time in days (95% CI)	Mean time in days (95% CI)	Mean time in days (95% CI)	Mean time in days (95% CI)
Traumatic brain injuries	0 (0–0)	66 (64–68)	326 (310–343)	722 (658–786)
Injuries excluding TBI	4 (4–4)	26 (24–27)	65 (57–73)	72 (47–97)
Psychiatric disorders and stress-related conditions	65 (64–66)	123 (121–126)	91 (83–99)	90 (64–115)
Musculoskeletal disorders	61 (60–61)	89 (87–91)	61 (55–68)	13 (4–21)
Miscellaneous	123 (122–123)	225 (222–228)	384 (367–401)	307 (259–354)
No work disability^b^	1,574 (1,573–1,575)	1,298 (1,294–1,303)	900 (878–921)	623 (560–688)

Abbreviation: TBI = traumatic brain injury.

Presented as mean time (95% CI).

a Detailed information on categories of cause of work disability and the analysis can be found in the eMethods and eTable 1.

b Days of no current work disability during the follow-up period of 5 years.

During 2008–2015, legislative changes introduced a 914-day cap on sickness benefits, forcing some individuals to discontinue benefit receipt despite persistent incapacity. Sensitivity analyses excluding this period yielded similar results (eFigure 2).

## Discussion

In this nationwide study, we examined long-term postinjury work disability risk across all TBI severities. We compared 98,256 patients with TBI treated in hospitals and open specialized care (including emergency departments) for varying TBI severities to a matched cohort of 981,191 non-TBI individuals. The risk of work disability was higher for individuals with TBI, regardless of their severity, compared with the matched cohort. The probability increased with TBI severity: from 9% in the least severe group (TBI A: not hospitalized or hospitalized ≤2 days), to 29% in TBI B (hospitalized ≥3 days, no neurosurgery), and 43% in TBI C (neurosurgery). Furthermore, we found that preinjury sociodemographic and medical factors, such as older age, female sex, lower education, substance use disorders, and psychiatric disorders were differently associated with work disability across the TBI groups.

The highest probability was seen in the more severe TBI groups (TBI B and C), consistent with previous research.^23^ However, unlike previous studies that measured return to work as a binary outcome,^6,9^ we assessed work disability as a recurring event and demonstrated that all 3 TBI groups had an elevated risk of transitioning to work disability during the entire 5-year follow-up, with an average duration of 526 days in TBI A to 1,201 days in TBI C.

TBI B and C peaked in their transition to work disability rates shortly after the TBI (around 30 days), whereas TBI A, exhibited their highest probability between 2 to 3 years postinjury. This delayed transition may reflect a progressive chronic state where symptoms are worsened over time or more likely may result from prolonged strain caused by returning to work while experiencing symptoms.^24^ The delayed transition in TBI A could be partly augmented over time by sociodemographic and medical factors, which also are associated with work disability in the general population, as seen in our matched cohort and in previous studies.^25^ Importantly, not all episodes of work disability after TBI were attributable to TBI-related diagnoses. As shown in Table 4, a substantial proportion of work disability was due to non-TBI medical conditions, and the 4 groups differed at baseline regarding somatic and psychiatric comorbidities, highlighting the possibly heterogeneous etiology of work disability in this population. Hence, it is important to assess symptoms related to the TBI such as fatigue and headache for patients who seek health care in the postacute phase, while also evaluating how sociodemographic and other medical factors that may affect health and function.

We evaluated multiple sociodemographic and medical factors as predictors of transitions to work disability after TBI. Older age consistently predicted higher risk across all severity groups, likely related to better outcome from greater neuroplasticity and fewer age-related changes. Educational level showed a different pattern: in the non-TBI cohort, higher education was a strong protective predictor, while in TBI A it was smaller but remained significant, and in TBI B and C it did not reach statistical significance. Educational level is considered a proxy for cognitive reserve, facilitating recovery and adaptation after injury.^26^ In addition, educational level can reflect different socioeconomic status,^27^ with impact on recovery, and difference at work with different flexibility.^28^ In more severe TBI, the dominant injury may overshadow the influence of education, and smaller sample sizes in these groups reduce power to detect modest associations. This gradient suggests that education is an important predictor in the general population, but its influence becomes less pronounced as TBI severity increases. Previous studies on both age and education and return to work have been inconsistent,^6,10^ which may partly reflect differences in sample size and severity composition.

There were few sociodemographic and medical factors associated with the probability of transitioning to work disability in TBI C. This could suggest that the highest contributing factor to work disability was the impact of severe functional impairment and that this overshadowed the influence of other patient-specific factors. This was also reflected in the cause of work disability within this group, where TBI was the most prevalent diagnosis, compared with miscellaneous reasons in the other groups. This underlines the need to provide individualized, specialized rehabilitation for patients with more severe TBI that identifies and delivers interventions for the specific disabilities at hand.

In the reverse transition, from work disability to nonwork disability (i.e., not receiving benefits), the probability was lower for all TBI groups and decreased with injury severity. Most sociodemographic and medical factors showed an inverse association: Factors associated with a higher probability of transitioning to work disability had the opposite association on transitioning to nonwork disability, as could be expected. However, having a blue-collar job was associated with a higher probability of transitioning to nonwork disability in all groups except TBI C, where the injury severity might diminish the importance of occupational factors. Our results in groups A and B could suggest that, despite being linked to increased work disability, manual labor may also be easier to return to, possibly because of better work adaptation or the nature of the tasks involved.

Although sex differences have been reported in previous studies, findings have not been consistent.^6,10^ European and American studies suggest that sex may play a more significant role than in Asian studies, potentially reflecting societal and cultural differences.^10^ In our study, being female was associated with a higher probability of transitioning to work disability for all groups except the most severe, which could be due to its smaller size, or the greater injury severity reducing the influence of sex. On the other hand, the sex differences seen in the other TBI groups, as well as in non-TBI, could reflect higher overall rates of work disability in women due to conditions in female-dominated occupations and greater domestic responsibilities.^29^ However, there may be specific challenges for women after a TBI because of different pathologic responses to injury in the brain, hormonal influence, psychosocial factors or differences in presentation and report of symptoms, warranting more research.^30^

In this study, we examined medically certified work disability, defined as physician-verified reductions in work capacity because of disease or injury. This outcome offers a closer approximation of health-related functional impairment than return-to-work, which is also influenced by workplace accommodations, employer practices, and labor market conditions. However, this study cannot verify if an individual who no longer receives sickness benefit (i.e., displays no work disability) has returned to the same occupation or to a different one. The study benefited from nationwide register data, including nearly 100,000 individuals with TBI across all severities and a 5-year follow-up period, where attrition was minimal compared to most long-term TBI studies.^12^ The inclusion of a matched cohort of almost 1 million individuals further allowed comparisons of work disability and associated factors with those observed in the general population.

We adjusted for multiple sociodemographic and medical factors, but residual confounding cannot be excluded. Preinjury conditions were captured in broad diagnostic categories, with limited clinical detail but allowing group-level comparisons of the somatic burden between TBI and non-TBI individuals. Disabilities resulting in work incapacity were captured through the work disability in the 3 years before enrollment variable. Misclassification bias may occur when using International Classification of Diseases codes,^31^ although Sweden's patient register, including concussion diagnoses, has high validity.^19,32^ The legislative reform 2008 capping sickness benefits at 914 days likely contributed to the visible dent in transition probabilities around 2.5 years, when some individuals were forced to exit benefits despite ongoing incapacity.^22^ However, sensitivity analyses excluding this period showed comparable results, suggesting robustness.^20^ We could not track work disability in individuals ineligible for sickness benefits, more often unemployed, which likely explains why being employed at the time of TBI predicted later work disability, in contrast to some earlier studies.^6,10^ Still, as most working-age Swedes are covered by national insurance, transitions to work disability remain a good indicator of health-related loss of work capacity. Absolute risks, however, may not be directly comparable internationally, although the associations with sociodemographic and occupational factors are likely generalizable to countries with similar sociocultural contexts regardless of insurance design. A further methodologic limitation is the substantial imbalance in group sizes, with 93% of cases classified as TBI A and <1% as TBI C. This distribution reflects real-world TBI epidemiology but results in reduced statistical precision for the most severe group, reflected by wider CIs.

Our use of ≤48 hours of hospital stay as a proxy for milder TBI is supported by the Scandinavian guidelines, which emphasize that patients recovering quickly and without significant CT findings can be discharged within this time frame.^33^ However, length of stay can also reflect comorbidities, resource availability, and local practices.^34^ Using neurosurgical intervention as a proxy for severe TBI is supported by previous research,^31^ and standard treatment algorithms, where severe injury with loss of consciousness typically requires surgery for intracranial monitoring or hematoma evacuation.^35^ Still, the lack of detailed clinical data such as GCS scores prevented standardized severity grading. This limits comparisons with studies restricted to mild or moderate-to-severe TBI, but by including all cases, we view severity as a continuum of pathology.

We found that TBI, across all severity groups, was associated with persistently increased probability of transitioning to work disability. Higher age, female sex, and psychiatric and substance use disorders were linked to higher risk in all but the most severe group, where only age remained predictive. These findings emphasize the need for long-term, individualized rehabilitation that addresses both injury-related impairment and sociodemographic or comorbid factors.

## Glossary

HR: hazard ratio
IQR: interquartile range
MiDAS: Micro-Data for Analysis of the Social Insurance System
NPR: National Patient Register
TBI: traumatic brain injury
